# Identification and Pathogenicity Analysis of *Huaxiibacter chinensis* Qf-1 in Mink (*Neogale vison*)

**DOI:** 10.3390/microorganisms13071604

**Published:** 2025-07-08

**Authors:** Yao Chen, Haotian Cai, Xiaoyang Wu, Xibao Wang, Yongquan Shang, Qinguo Wei, Weilai Sha, Yan Qi, Shuli Liu, Honghai Zhang

**Affiliations:** 1 College of Life Sciences, Qufu Normal University, Qufu 273165, China; chenyao@qfnu.edu.cn (Y.C.); caihaotian0915@163.com (H.C.); wuxiaoyang1988@126.com (X.W.); wangxibao1995@163.com (X.W.); yongquanshang@163.com (Y.S.); qgwei2008@163.com (Q.W.); shaweilai@163.com (W.S.); qiyan94163@126.com (Y.Q.); 2 Zhonghuan Shengda Environmental Technology Group (Qingyun) Co., Ltd., Dezhou 253000, China; 13910335155@163.com

**Keywords:** *Neogale vison*, *Huaxiibacter chinensis* Qf-1, culturomics, genome, pathogenicity

## Abstract

Mink (*Neogale vison*) is a commercially farmed animal of global importance. However, disease outbreaks during farming not only cause significant economic losses but also substantially increase the risk of zoonotic infections. The identification and characterization of pathogenic bacteria remain a major bottleneck restricting the development of healthy and sustainable mink farming. In this study, an LB medium was used to isolate a pale-white, rod-shaped, Gram-negative bacterial strain, Qf-1, from minks with pneumonia. Based on morphological characteristics, biochemical properties, 16S rRNA gene sequencing, and average nucleotide identity (ANI) analysis, strain Qf-1 was identified as *Huaxiibacter chinensis* Qf-1. Under laboratory conditions, *H. chinensis* Qf-1 induced typical pneumonia symptoms in Kunming mice. Furthermore, whole-genome sequencing of *H. chinensis* Qf-1 revealed its genome to be 4.77 Mb and to contain a single chromosome and one plasmid. The main virulence genes of *H. chinensis* Qf-1 were primarily associated with *flgB, flgC, flgG, aceA, hemL, tssC1, csgD, hofB, ppdD, hcpA*, and *vgrGA*, functioning in motility, biofilm formation, colonization ability, and secretion systems. Our findings contribute to a better understanding of their pathogenic mechanisms, thereby laying a theoretical foundation for further investigation into the complex interactions between gut microbiota and the host.

## 1. Introduction

The mink (*Neogale vison*), belonging to the order Carnivora, family Mustelidae, and genus *Neovison*, is an economically important fur-bearing animal that is widely farmed in Europe, North America, and China [1]. However, due to the lack of standardized breeding requirements and protocols, the occurrence and spread of bacterial diseases in minks have severely threatened the healthy development of the mink farming industry, resulting in significant economic losses. Bacterial diseases in minks are characterized by mixed infections, easily confused clinical symptoms, difficulties in pathogen isolation, and increasing antibiotic resistance [2,3,4].

At present, research on viral diseases in minks has been relatively thorough and comprehensive. Mink enteritis virus (MEV), Aleutian mink disease virus (AMDV), and canine distemper virus (CDV) have emerged as the three major viral pathogens posing significant threats to the mink farming industry. These viruses are responsible for causing highly contagious viral enteritis, Aleutian disease, and canine distemper in minks, respectively [5,6]. Significant breakthroughs have been achieved in research on mink viruses, particularly in areas such as taxonomic classification, genome structure, gene function, pathogenic mechanisms, and vaccine development. These advances have played a pivotal role in promoting the ecological prevention and control of viral diseases in minks [7].

Compared with viral diseases, research on bacterial diseases in minks began relatively late. With the rapid expansion of mink farming, bacterial outbreaks have occurred with increasing frequency, causing irreversible impacts on the industry. In recent years, the pathogenesis and ecological prevention of bacterial diseases in minks have gradually become research hotspots. Various bacterial pathogens have been detected in mink hosts, including *Escherichia coli*, *Pseudomonas aeruginosa*, *Streptococcus canis*, *Streptococcus dysgalactiae*, *Staphylococcus delphini*, *Staphylococcus aureus*, *Staphylococcus schleiferi*, *Pasteurella multocida*, *Staphylococcus intermedius*, *Staphylococcus aureus*, and *Klebsiella pneumoniae* [8,9]. However, existing studies have primarily focused on the antimicrobial resistance of these opportunistic pathogens, with limited investigation into their specific pathogenic mechanisms [2,10].

In summary, the isolation and identification of pathogenic bacteria are critical steps for the precise prevention and control of mink diseases. This study focused on minks affected by bacterial pneumonia and employed culturomics and genomics approaches to isolate and identify opportunistic bacterial pathogens, as well as to analyze their pathogenicity. The findings of this research will not only contribute to the establishment of stable experimental models between pathogens and mink hosts, providing a foundation for further investigation into their virulence mechanisms, but they will also offer theoretical support for the precise prevention and control of bacterial diseases in mink farming.

## 2. Materials and Methods

### 2.1. Sample Collection

In December 2023, the Youan Mink Breeding Co., Ltd. in Qingdao, China, housed a total of 3200 minks, of which 74 displayed typical symptoms of pneumonia, including respiratory distress, elevated body temperature, anorexia, lethargy, and the presence of red, bubble-like discharge from the nostrils. The mink exhibiting signs of pneumonia were isolated and treated at the farm with intramuscular injections of florfenicol and doxycycline. Following treatment, no instances of transmission were observed, and all 74 minks fully recovered. Therefore, the feces of mink exhibiting symptoms of pneumonia were collected into 5 mL centrifuge tubes pre-filled with glycerol and transported back to the laboratory on the same day of collection.

### 2.2. Bacterial Isolation

The fecal samples were collected from mink with pneumonia. The samples were diluted 10-fold with an appropriate volume of normal saline, and subsequently serially diluted 10-fold up to a dilution factor of 10^−9^. From each dilution, 30 µL of the bacterial suspension was cultured on an LB liquid medium at 35 °C for 24 h using the dilution and spread plate method. After overnight incubation, single colonies with different morphologies and sizes were selected and further purified by streaking using the three-zone streaking technique. This purification process was repeated 4–5 times to ensure strain purity. The purified bacterial isolates were preserved at −80 °C in 30% (*v*/*v*) glycerol for future use.

### 2.3. Morphological, Physiological, and Biochemical Analysis of Strain QF-1

During the bacterial isolation and purification stage, the morphological characteristics of individual colonies were visually examined and documented through photography. Gram staining of strain Qf-1 was performed using a Gram staining kit (G1065, Servicebio, Wuhan, China). The cellular morphology of strain Qf-1 was observed using both scanning electron microscopy (SEM, JOUQDSM-840, JEOL, Akishima, Japan) and transmission electron microscopy (TEM, JEM-1200EX, JEOL, Japan), following the protocol described by Zhang et al. [11].

The growth of strain Qf-1 was monitored by measuring the optical density at 600 nm (OD_600_) every 2 h. A standard growth curve was plotted based on these measurements, following the method reported by Tsutsuki H. et al. [12]. A linear regression model was applied in Origin (2021; OriginLab Corp., Northampton, MA, USA) to generate the standard growth curve and calculate the corresponding R^2^ value. To ensure data transparency and reproducibility, both the growth curve and the standard curve were established using three biological replicates and three technical replicates.

The biochemical and physiological characteristics of strain Qf-1 were evaluated using the Biolog Gen III MicroPlate system (Biolog, Hayward, CA, USA) according to the manufacturer’s instructions. The strain was first cultured in an LB liquid medium at 33 °C for 24 h. Subsequently, the bacterial suspension was adjusted to 98% turbidity using Biolog Fluid A. A volume of 100 µL of the bacterial suspension was inoculated into each well of the Gen III MicroPlate. After incubation at 33 °C for 24 h, the results were automatically recorded at 600 nm using the standardized MicroStation™ system (Biolog Inc., Hayward, CA, USA). Two wells were designated as negative and positive controls, indicated by colorless and purple reactions, respectively.

### 2.4. 16S rRNA Gene Sequencing and Construction of Phylogenetic Tree

The 16S rRNA gene of strain Qf-1 was amplified by PCR using the universal primers 27F and 1492R [13]. The PCR products were examined by electrophoresis on a 2% agarose gel. Purification of the amplified products was carried out using the AxyPrep DNA Gel Extraction Kit (Axygen Biosciences, Union City, CA, USA), according to the manufacturer’s instructions. The purified 16S rRNA gene fragments were subjected to paired-end sequencing using the Sanger method. The assembled 16S rRNA gene sequence was subsequently submitted to the National Center for Biotechnology Information (NCBI) database.

Preliminary identification of strain Qf-1 and comparison with closely related type strains were carried out using the EzBioCloud server (http://www.ezbiocloud.net, accessed on 8 October 2024) [14] and the BLAST (v2.2.25) tool provided by the NCBI database (http://www.ncbi.nlm.nih.gov, accessed on 8 October 2024). Phylogenetic analysis was performed using MEGA (vX) [15], and the phylogenetic tree was reconstructed using the neighbor-joining (NJ) algorithm [16]. The Kimura two-parameter model [17] was applied for the calculation of evolutionary distances. The robustness of the phylogenetic tree topology was evaluated by 1000 bootstrap replications [18].

### 2.5. Genome Sequencing and Analysis of Average Nucleotide Identity (ANI)

The EZ-10 Spin Column Bacterial Genomic DNA Isolation Kit (B610423-0050, Sangon Biotech, Shanghai, China) was used to extract genomic DNA from strain Qf-1. A whole-genome shotgun (WGS) sequencing strategy was employed, with libraries of varying insert sizes constructed for both second-generation and third-generation sequencing. Illumina NGS and single-molecule long-read sequencing were performed on the Illumina platform at Paisonor BioTech Co., Ltd. (Shanghai, China). Raw short-read data were quality-filtered using fastp (v0.24.1) [19]. Long reads were assembled de novo with Unicycler (v0.5.1) [20], Flye (2.9.5) [21], Hifiasm (0.25.0) [22], and Necat (v0.0.1_update20200803) [23], and assemblies were polished with Pilon (v1.24) [24] using high-quality Illumina reads. Assembly completeness and contamination were assessed with CheckM (v1.2.3) [25]. Average nucleotide identity (ANI) was calculated via the ANI/AAI-Matrix online tool (Kostas Laboratory; http://enve-omics.ce.gatech.edu/g-matrix/, accessed on 10 October 2024, North Avenue, Atlanta) [26].

For functional annotation, genome annotation was performed using the genome analysis tool available at the Type Strains Genome Database (https://gctype.wdcm.org/, accessed on 14 October 2024), in combination with the Clusters of Orthologous Groups (COG) database [27]. Additionally, functional annotation was conducted using the eggNOG online server (http://eggnog5.embl.de/#/app/home, accessed on 13 October 2024) [14]. KEGG Orthology assignments were generated via KAAS (BlastKOALA; https://www.kegg.jp/blastkoala/, accessed on 14 October 2024) [28]. Virulence-associated genes were identified using the VFDB online server [29]. Carbohydrate-active enzymes (CAZymes) were annotated via the dbCAN3 meta server (https://bcb.unl.edu/dbCAN2/, accessed on 15 October 2024) [30]. Protein secretion systems were predicted with MacSyFinder (2.1.4) [31], and type III secretion effectors were identified using EffectiveT3 (Version 3.0) [32]. Finally, two-component regulatory systems were cataloged based on Pfam domain annotations.

### 2.6. Bacterial Challenge Infection Assay

Before conducting the bacterial challenge infection assay, the Qf-1 strain was activated. First, Qf-1 was brought to room temperature, and a loop was used to pick up the bacterial suspension, which was streaked onto an LB liquid medium for incubation at 35 °C for 8 h. Single colonies were then picked and inoculated into 100 mL of the LB liquid medium, followed by incubation in a shaking incubator (35 °C, 150 rpm) for 8 h. Subsequently, a passage culture was prepared by transferring 1 mL of the bacterial suspension to 100 mL of a fresh LB liquid medium and cultured until the OD_600_ = 0.40 (equivalent to 1.0 × 10^8^ CFU/mL), indicating that the bacterial culture was in the logarithmic growth phase.

For the infection experiment, 3-week-old Kunming mice purchased from Shandong Pengyue Laboratory Animal Technology Co., Ltd. (Jinan, China), were used. After 3 days of standard feeding in the laboratory, 0.2 mL of the Qf-1 bacterial suspension (OD_600_ = 0.40) was injected into the peritoneum of the mice. The mice were monitored every 2 h for changes in their general condition, and their body weight was recorded every 24 h. After 5 days of observation, the mice were euthanized, and their lung tissues were collected for hematoxylin and eosin (HE) staining, which was carried out by Wuhan Servicebio Co., Ltd. (Wuhan, China).

## 3. Results

### 3.1. Morphological Characteristics of Pathogenic Bacterial Strain of Qf-1

**Figure 1:**
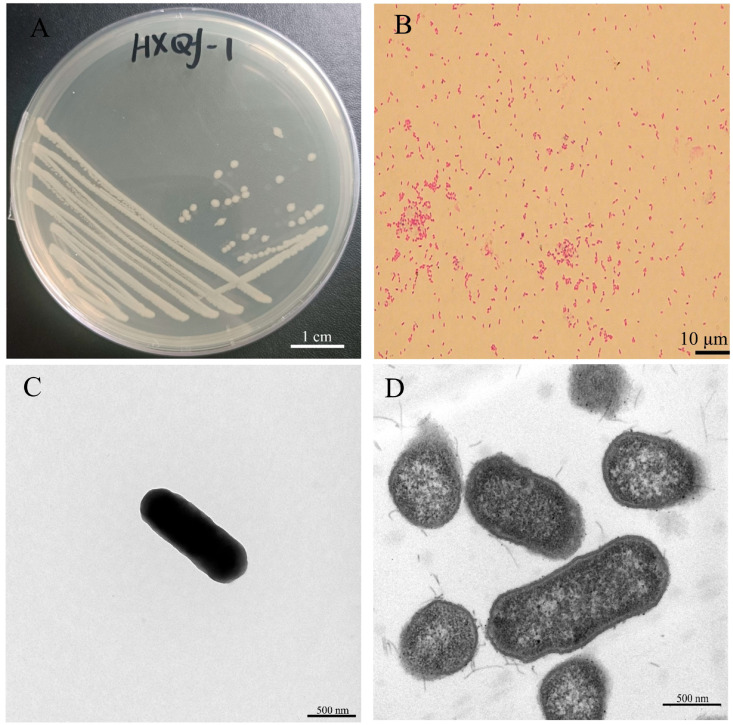
Morphological characteristics of Qf-1. (**A**) Qf-1 colonies on LB culture medium (bar = 1 cm). (**B**) Gram staining of QF-1 (bar = 10 µm). (**C**) Morphology of XP-2 observed by SEM (bar = 500 nm). (**D**) Morphology of XP-2 observed by TEM (bar = 500 nm).

The Qf-1 strain was identified as Gram-negative (Figure 1A) and exhibited a light white coloration on the LB culture medium, forming circular colonies with diameters ranging from 0.80 to 1.20 mm (Figure 1B). SEM (Figure 1C) and TEM (Figure 1D) revealed that the cells were rod-shaped, measuring approximately 1.20–1.79 µm in length and 0.72–0.98 µm in width, and lacked flagella.

### 3.2. Growth Curve and the Standard Growth Curve of Qf-1

**Figure 2:**
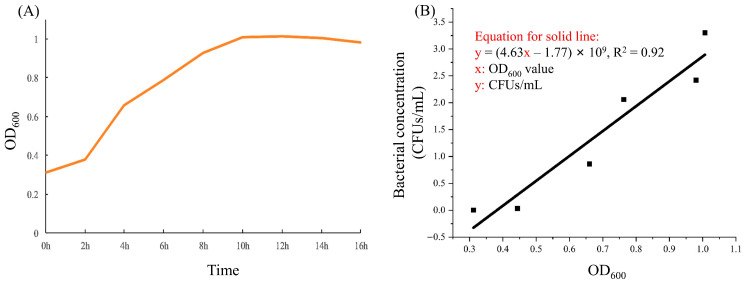
Growth curve and the standard growth curve of Qf-1. (**A**) The growth curve of Qf-1. (**B**) The standard growth curve of Qf-1.

As shown in Figure 2, the growth curve indicates that Qf-1 enters the logarithmic growth phase at 2 h, when OD_600_ is 0.40. From 2 to 10 h, Qf-1 remains in the logarithmic growth phase, and Qf-1 enters the stationary phase after 10 h when OD_600_ reaches 1.00. The standard curve for Qf-1 is denoted as y = (4.63x − 1.77) × 109, R^2^ = 0.92.

### 3.3. Molecular Identification and Phylogenetic Analysis

**Figure 3:**
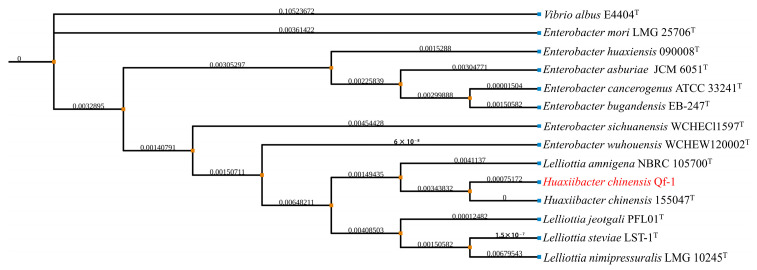
Neighbor-joining phylogenetic tree based on 16S rRNA gene sequences. Percentage bootstrap values above 50% (1000 replicates) are shown at branch nodes. Bar = 0.020, substitutions per nucleotide position. *Vibrio albus* E4404^T^ was used as an outgroup.

The partial 16S rRNA gene sequence of strain Qf-1 was 1392 bp. A neighbor-joining phylogenetic tree was constructed based on 16S rRNA gene sequences, showing the phylogenetic positions of strain Qf-1 and the related *Huaxiibacter* species. The strain Qf-1 was most closely related to *H. chinensis* 155047^T^ with 99.93% similarity (Figure 3). An analysis of the average nucleotide identity (ANI) between strain Qf-1 and *H. chinensis* 155047^T^ revealed that the similarity was 98.77% (Table S1).

### 3.4. Biochemical Characterization of Qf-1 Using Biolog Gen III Microtest System

Strain Qf-1 exhibited its ability to react positively to 47 (50.00%), weakly positive to 16 (17.02%), and negatively to 29 (30.85%) out of the 94 different physiological and biochemical traits. Qf-1 grew on a wide range of sugars (e.g., D-Turanose, D-Galactose, L-Rhamnose, Sucrose, Gentiobiose, and α-D-Glucose), hexose-PO4 (e.g., D-Glucose-6-PO4 and D-Fructose-6-PO4), and amino acids (e.g., D-Glucuronic Acid, D-Gluconic Acid, L-Glutamic Acid, Acetic Acid, Bromo-Succinic Acid, and L-Lactic Acid), as shown in Table 1.

Based on the high similarity between 16S rRNA gene sequences of strain Qf-1 and *H. chinensis* 155047^T^, as well as the chemical characterization of strain Qf-1, it was designated as *H. chinensis* Qf-1.

### 3.5. Observations of HE Staining and Bacterial Changes in Infected Tissue by H. chinensis Qf-1

**Figure 4:**
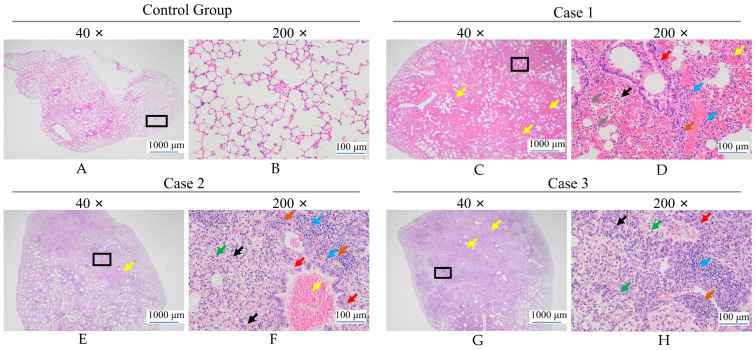
Histopathological observation of lung tissue in Kunming mice infected with *H. chinensis* Qf-1. Note: Extensive hemorrhage (yellow arrows); mild edema of bronchiolar epithelial cells (red arrows); mild infiltration of granulocytes (black arrows); numerous macrophages in the alveolar spaces (gray arrows); prominent peribronchiolar and perivascular lymphocytic (blue arrows); granulocytic (orange arrows) infiltration forming ring-like patterns; and proliferation of connective tissue (green arrows). The black box indicates the magnified area shown. Bar: (**A**,**C**,**E**,**G**) = 1000 µm; (**B**,**D**,**F**,**H**) = 100 µm.

Following HE staining, there were no pathological alterations in the control group, and the cellular morphology remained intact and uniform (Figure 4A,B). In the experimental groups, infection with *H. chinensis* Qf-1 in Kunming mice induced inflammatory responses in the lung tissues. The primary pathological changes included extensive hemorrhage (Figure 4C–G; yellow arrows), edema of bronchial epithelial cells (Figure 4D,F,H; red arrows), proliferation of connective tissue (Figure 4F,H; green arrows), and infiltration of granulocytes (Figure 4D,F,H; black and orange arrows). Additionally, the presence of macrophages (Figure 4D; gray arrows) and lymphocytes (Figure 4D,F,H; blue arrows) was observed in the pulmonary tissue.

### 3.6. Whole-Genome Analyses of Strain H. chinensis Qf-1

As shown in Table 2, the clean reads of *H. chinensis* Qf-1 were 9,768,592 bp. The genome size of *H. chinensis* Qf-1 was 4.77 Mb with a GC content of 48.99%. There were approximately 4445 protein-coding genes, and 4149, 2916, 3573, and 3841 genes were annotated against the Evolutionary Genealogy of Genes: Non-Supervised Orthologous Groups (eggNOG), Kyoto Encyclopedia of Genes and Genomes (KEGG), Gene Ontology (GO), and Swiss-Prot databases, respectively. In addition, 80, 7, 192, 143, and 7 genes were annotated against the two-component signaling or regulatory system (TCS) (Table S2), Clustered Regularly Interspaced Short Palindromic Repeats (CRISPRs), Virulence Factor Database (VFDB) (Table S3), Comprehensive Antibiotic Resistance Database (CARD) (Table S4), and type III secretion system (T3SS) (Table S5), respectively.

**Figure 5:**
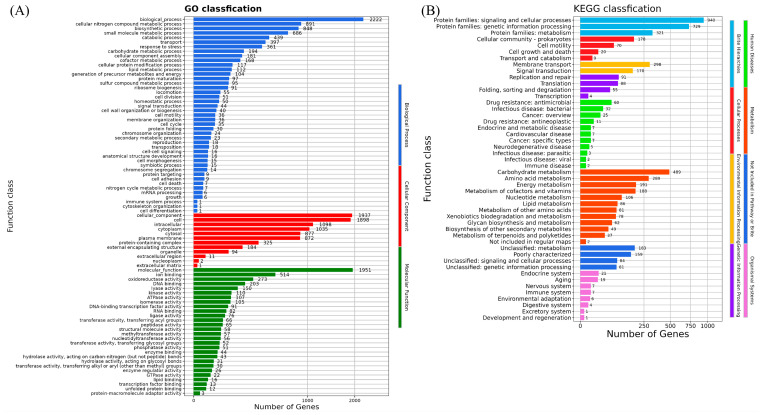
The genome functional annotation of *H. chinensis* Qf-1 against the GO and KEGG databases (**A**), the GO annotation of *H. chinensis* Qf-1; (**B**) the KEGG annotation of *H. chinensis* Qf-1.

Inorganic ion transport and metabolism, transcription, carbohydrate transport and metabolism, amino acid transport and metabolism, cell motility, coenzyme transport and metabolism, replication, recombination, and repair were revealed by the genomic functional annotation of *H. chinensis* Qf-1 against the eggNOG database (Figure S1). Moreover, the cellular component, molecular function, and biological process terms of XH1 were also classified by genome functional annotation against the GO database (Figure 5A). Additionally, the human diseases, metabolism, not included in the pathway of Brite, organismal systems, Brite hierarchies, cellular processes, environmental information processing, and genetic information processing terms of *H. chinensis* Qf-1 were also classified by genome functional annotation against the KEGG database (Figure 5B).

### 3.7. Pathogenic Potential Analysis of Strain H. chinensis Qf-1

As shown in Figure 2, 34 virulence genes have different biological functions in *H. chinensis* Qf-1, mainly including the functions of CARD, T3SS, and TNSS. Among them, *tssM1*, *clpV1*, *tssK1*, *hcpA*, and *vgrGA* belong to T6SS coding genes. In addition, *flgB*, *flgC*, *flgD*, *flgE*, *flgF*, *flgG*, *flgK*, *flgL*, and *flgL* are flagella genes of the flagella system. Furthermore, *hofB* and *ppdD* are the coding genes of type IV fimbriae.

### 3.8. Genome Assembly Completion Mapping of Strain H. chinensis Qf-1

**Figure 6:**
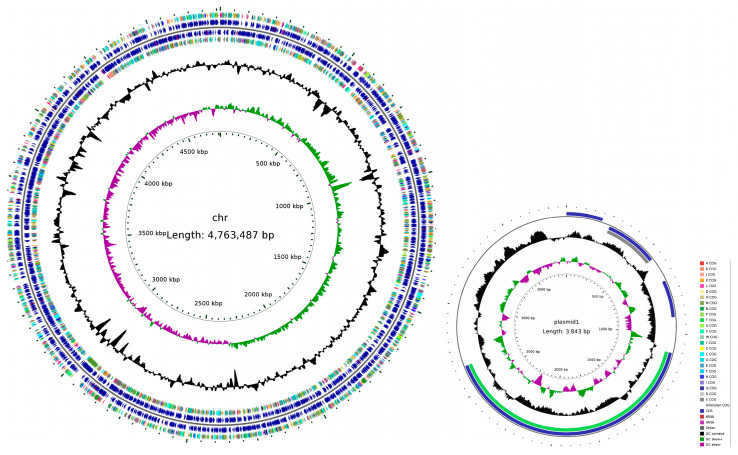
Circular representation of the *H. chinensis* Qf-1 genome. From the inside to the outside, the first circle represents the scale; the second circle represents GCSkew; the third circle represents GC content; the fourth and seventh circles represent COGs, to which each CDS belongs; and the fifth and sixth circles represent the positions of CDS, tRNA, and rRNA on the genome.

The circle map of *the H. chinensis* Qf-1 genome was shown in Figure 6, which includes one chromosome (4,763,487 bp) and one plasmid (3843 bp). Circle 1 (from inside to outside) represents the scale; Circle 2 represents GCSkew; Circle 3 represents GC content; Circle 4 and Circle 7 represent COGs, to which each CDS belongs; and Circle 5 and Circle 6 represent the positions of CDS, tRNA, and rRNA on the genome.

## 4. Discussion

In this study, based on morphological observations, physiological and biochemical characteristics, phylogenetic analysis, and average nucleotide identity (ANI) calculations, strain Qf-1 was identified as *H. chinensis* Qf-1. This is the first report of *H. chinensis* Qf-1 isolated from the intestinal tract of mink. In the infection experiment, *H. chinensis* Qf-1 was capable of inducing typical pneumonia symptoms in Kunming mice (Figure 4). In addition, whole-genome sequencing analysis revealed that *H. chinensis* Qf-1 harbors multiple virulence factors, providing valuable reference data for the clinical prevention and control of epizootic diseases in mink. *H. chinensis* Qf-1 is a pathogenic bacterium responsible for the pneumonia disease of mink, suggesting that *H. chinensis* Qf-1 may be a natural component of the gut microbiota of mink, which requires further validation with a larger sample size in future studies. However, it should be noted that dysbiosis of the gut microbiota may increase susceptibility to pneumonia [34]. In this context, our study focused on the gut microbiota of mink, further isolating and identifying intestinal bacteria from pneumonic mink to identify opportunistic pathogens potentially associated with pneumonia. Therefore, we chose mink feces for bacterial isolation and identification. Additionally, the gut can influence distal pulmonary responsiveness and inflammation via microbial metabolites, immune cell trafficking, and neuroendocrine signaling pathways [35,36,37,38]. Moreover, intraperitoneal injection is a commonly used technique in laboratory rodents [39]. As a result, in this study, we chose intraperitoneal injection rather than respiratory administration.

*H. chinensis* 155047^T^ was first reported by He et al. in 2022 and was isolated from the sputum of a patient in China [33]. The strain is positioned within the *Enterobacter–Leclercia–Lelliottia–Pseudenterobacter* lineage; however, both its average nucleotide identity (ANI) and average amino acid identity (AAI) values fall below the genus-level thresholds [40], indicating that it represents a novel genus within this lineage. Based on its genotypic and phenotypic characteristics, the authors proposed the name *Huaxiibacter* for the novel genus and *H. chinensis* for the novel species. The type strain is 155047^T^ [33].

In terms of morphology, cells of *H. chinensis* Qf-1 are Gram-negative (Figure 1A) and exhibit a light white coloration, forming circular colonies with diameters ranging from 0.80 to 1.20 mm (Figure 1B), which is similar to *H. chinensis* 155047^T^. Secondly, in terms of physiological and biochemical characteristics, acid is produced when *H. chinensis* Qf-1 is cultured with N-Acetyl-D-Galactosamine, D-Galactose, L-Rhamnose, Gentiobiose, α-D-Glucose, D-Cellobiose, D-Sorbito, D-Mannose, D-Trehalose, D-Maltose, Melibiose, D-Mannitol, a-D-Lactose, D-Fructose, D-Salicin, and L-Fucose but not in the presence of D-Fucose, myo-Inositol, and D-Arabitol. The carbon source utilization profile of *H. chinensis* Qf-1 is similar but not identical to that of *H. chinensis* 155047^T^, which may be attributed to evolutionary divergence between the strains and differences in the detection kits used. Based on morphological characteristics and physiological and biochemical tests alone, the taxonomic placement of strain Qf-1 could not be conclusively determined. Therefore, whole-genome sequencing was performed for strain Qf-1, and a complete genome map was constructed. Firstly, phylogenetic analysis revealed that *H. chinensis* Qf-1 is most closely related to *H. chinensis* 155047^T^, with a similarity of 99.93% (Figure 3). Second, the average nucleotide identity (ANI) between strain Qf-1 and *H. chinensis* 155047^T^ was calculated to be 98.77% (Table S1), which exceeds the commonly accepted threshold for species delineation [40]. These molecular identification results provide strong evidence that strain Qf-1 isolated in this study belongs to the species *H. chinensis*. It is worth noting that, owing to the clarity provided by the phylogenetic analysis and ANI calculation, the taxonomic status of strain Qf-1 could be reliably determined without the need for additional comprehensive characterization. In contrast, the original study describing *H. chinensis* 155047^T^ involved more extensive analyses, including assessments of motility, anaerobic growth capacity, and fatty acid composition, as it was a newly discovered species at the time.

Research on *H. chinensis* remains in its early stages. Notably, *H. chinensis* 155047^T^ has not been explored for its pathogenic potential, focusing exclusively on its taxonomic classification [33]. Furthermore, using 16S rRNA amplicon sequencing and culturomics, the study revealed the diversity of gut microbiota in hibernating bats and successfully isolated and cultured *H. chinensis*, which is potentially pathogenic to humans [41]. Therefore, to investigate the pathogenicity of *H. chinensis*, we first employed H&E staining in this study to assess the pathogenicity of *H*. *chinensis* Qf-1. The results demonstrated that *H*. *chinensis* Qf-1 can induce typical pneumonia symptoms in Kunming mice, including extensive hemorrhage, edema of bronchial epithelial cells, proliferation of connective tissue, and infiltration of granulocytes, while the presence of macrophages and lymphocytes is observed in the pulmonary tissue. Therefore, based on the results of H&E staining, we performed whole-genome sequencing of *H*. *chinensis* Qf-1 and constructed its complete genome map. Furthermore, genes potentially associated with pathogenicity in *H*. *chinensis* Qf-1 were analyzed. The genome of *H*. *chinensis* Qf-1 is 4.77 Mb in size and consists of one chromosome and one plasmid. Functional annotation of the genome revealed that its pathogenic potential is primarily associated with genes encoding flagella, the type III secretion system (T3SS), type IV pili, and the type VI secretion system (T6SS), including the following key components: *flgB* (chr_1651), *flgC* (chr_1652), *flgG* (chr_1656), *aceA* (chr_223), *hemL* (chr_723), *tssC1* (chr_842), *csgD* (chr_1620), *hofB* (chr_713), *ppdD* (chr_722), *hcpA* (chr_844), and *vgrGA* (chr_845), where all encoding genes are virulence genes (Table 3)**.** Previous studies have shown that *flgB*, *flgC*, and *flgG* encode the basal body rod proteins of the flagellar system, which play a crucial role in bacterial motility and, consequently, influence the pathogenicity of the bacterium [42]. Inhibition of AceA can “freeze” *Acinetobacter baumannii* in a low-virulence viable but nonculturable (VBNC) state [43]. *hemL* influences the antibiotic resistance of *Salmonella enterica*, thereby affecting its pathogenicity [44]. CsgD is considered a central regulator controlling the transition of Salmonella between motile (planktonic) and sessile (biofilm) lifestyles, thereby influencing both its motility and biofilm formation capacity—factors that are critical determinants of its pathogenicity [45]. *hofB* plays a role in pilus formation, which initiates pathogen attachment, invasion, and biofilm formation [46]. Additionally, it is an important component of the type II secretion (T2SS) system, which contributes to bacterial survival and biofilm development [47]. Prepilin peptidase-dependent protein D (PpdD) is the major subunit of bacterial type IV pili (T4P), which are essential for host colonization and virulence in many Gram-negative bacteria. In enterohemorrhagic *Escherichia coli*, the T4P, known as hemorrhagic coli pili (HCP), facilitates cell adhesion, motility, biofilm formation, and signal transduction [48]. *VgrG* is an important virulence factor of the type VI secretion system in *Rahnella aquatilis*. VgrG mediates interactions between pathogenic bacteria and host macrophages, thereby influencing the pathogenicity of the bacteria [49]. In summary, the pathogenicity of *H. chinensis* Qf-1 may be associated with its motility, biofilm formation, colonization ability, and secretion systems.

## 5. Conclusions

Mink is an important species in China’s specialized economic animal farming. Like economically valuable crops, mink is susceptible to disease outbreaks during the farming process, which can result in significant economic losses and increase the risk of zoonotic disease transmission. Therefore, the isolation and identification of pathogenic bacteria remain a bottleneck in the prevention and control of mink-borne infectious diseases.

In this study, we isolated and identified the *H. chinensis* Qf-1 as an opportunistic pathogenic bacterium from pneumonia mink feces using culturomics. *H. chinensis* Qf-1 induced typical pneumonia symptoms in Kunming mice, indicating its potential pathogenicity and suggesting that it could pose a health risk to mink. Genomic sequencing and analysis further revealed that the pathogenicity of *H. chinensis* Qf-1 may be associated with its motility, biofilm formation, colonization ability, and secretion systems. Our findings expand the known diversity of pathogens responsible for animal-borne infectious diseases and contribute to a better understanding of their pathogenic mechanisms, thereby laying a theoretical foundation for further investigation into the complex interactions between gut microbiota and the host.

## Figures and Tables

**Table 1:** Characterization of strain Qf-1 based on the Biolog Gen III MicroPlate.

**Positive Reaction with the Following Substrates/Tests**		
D-Turanose	D-Glucuronic Acid	N-Acetyl-β-Dmannosamine
Glucuronamide	D-Saccharic Acid	N-Acetyl-D-Galactosamine ^*^
L-Histidine	D-Gluconic Acid	D-Glucose-6-PO4
L-Glutamic Acid	D-Galactose *	D-Fructose-6-PO4
Acetic Acid	Glycyl-L-Proline	Bromo-Succinic Acid
L-Rhamnose *	Citric Acid	D-Lactic Acid Methyl Ester
Sucrose	Gentiobiose *	N-Acetyl-D-Glucosami
L-Lactic Acid	α-D-Glucose *	b-Methyl-D-Glucoside
D-Cellobiose *	D-Sorbito *	D-Mannose
Methyl Pyruvate	L-Alanine	D-Trehalose
3-Methyl Glucose	D-Maltose *	L-Malic acid
Glycerol	Melibiose *	L-Aspartic Acid
L-Arginine	L-Serine	D-Mannitol *
Inosine	a-D-Lactose *	D-Galacturonic Acid
D-Fructose *	D-Salicin *	L-Galactonic Acid Lactine
Mucic Acid	L-Fucose *	
**Weak Positive Reaction with the Following Substrates/Tests**		
Pectin	Nalidixic Acid	L-Pyroglutamic Acid
Quinic Acid	PH6	c-Amino-Butyric Acid
Acetoacetic Acid	Vancomycin	β-Hydroxy-_D, L_-Butyric Acid
Dextrin	Sodium Lactate	a-Keto-Glutaric Acid
D-Raffinose	D-Malic Acid	
Tween40	Formic Acid	
**Negative Reaction with the Following Substrates/Tests**		
1%NaCl	Potassium Tellurite	Fusidic Acid
D-Fucose *	D-Serine	Minocycline
Propanoic Acid	Sodium Bromate	a-Hydroxy-Butyric Acid
4%NaCl	Guanidine HCl	Rifamycin SV
Myo-Inositol *	Aztreonam	a-Hydroxy-Butyric Acid
pH5	Troleandomycin	Lincomycin
D-Arabitol *	Sodium Butyrate	N-Acetyl-D-Galactosam
Gelatin	Lithium Chloride	D-Aspartic Acid
Stachyose	8%NaCl	p-Hydroxy-Phenylacetic Acid
D-Serine	Niaproof 4	

Note: *, described by He et al., 2022 [33].

**Table 2:** Qf-1 whole-genome sequencing result statistics.

Characteristic	Genome	Characteristic	Gnome
Size of raw reads (bp)	9,991,278	CRISPRs	7
Size of total reads (bp)	1,508,682,978	VFDB	192
Size of clean reads (bp)	9,768,592	CARD	143
Genome size (Mb)	4.77	T3SS	7
GC content (%)	48.99	Coding gene annotated	4445
Total gene size (bp)	4,168,185	Coding gene assigned to eggNOG	4149
rRNA	22	Coding gene assigned to KEGG	2916
tRNA	86	Coding gene assigned to GO	3573
ncRNA	130	Coding gene assigned to Swiss-Prot	3841
TCS	80		

**Table 3:** Qf-1 whole-genome sequencing results.

ORF Name	Gene Name	VF_ID	CARD	T3SS	TNSS
chr_223	*aceA*	VFG009263	-	TRUE	-
chr_232	*pgi*	VFG013531	-	TRUE	-
chr_532	*lpxC*	VFG013414	ARO: 3003574	-	-
chr_713	*hofB*	VFG042799	-	-	T4aP_pilB
chr_722	*ppdD*	VFG042800	-	-	T4aP_pilA
chr_723	*hemL*	VFG013618	-	TRUE	-
chr_769	*lpxA*	VFG013394	ARO: 3003573	-	-
chr_794	*tssM1*	VFG035488	-	-	T6SSi_tssM
chr_834	*clpV1*	VFG035568	-	-	T6SSi_tssH
chr_836	*tssK1*	VFG035613	-	-	T6SSi_tssK
chr_842	*tssC1*	VFG035762	-	TRUE	-
chr_844	*hcpA*	VFG041172	-		T6SSi_tssD
chr_845	*vgrGA*	VFG035855	-	-	T6SSi_tssI
chr_875	*phoE*	VFG043568	ARO: 3004122	-	-
chr_1031	*acrB*	VFG049136	ARO: 3000216	-	-
chr_1032	*acrA*	VFG049125	ARO: 3004042	-	-
chr_1081	*fimF*	VFG042684	-	TRUE	-
chr_1471	*gspE*	VFG007101	-	-	T2SS_gspE
chr_1473	*outG*	VFG040912	-	-	T2SS_gspG
chr_1532	*msbA*	VFG013253	ARO: 3003950	-	-
chr_1569	*ompA*	VFG043544	ARO: 3005044	-	-
chr_1620	*csgD*	VFG045791	-	TRUE	-
chr_1651	*flgB*	VFG043022	-	-	Flg flgB
chr_1652	*flgC*	VFG043075	-	-	Flg flgC
chr_1653	*flgD*	VFG043024	-	TRUE	-
chr_1654	*flgE*	VFG043077	-	TRUE	-
chr_1655	*flgF*	VFG043078	-	TRUE	-
chr_1656	*flgG*	VFG043079	-	TRUE	Flg flgC
chr_1660	*flgK*	VFG043083	-	TRUE	-
chr_1661	*flgL*	VFG043032	-	TRUE	-
chr_1672	*fabG*	VFG038840	ARO: 3004049	-	-
chr_1708	*phoQ*	VFG021077	ARO: 3007203	-	-
chr_1709	*phoP*	VFG000475	ARO: 3003585	-	-
chr_1815	*hemR*	VFG012601	-	TRUE	-

Note: ORF name, the ORF name of *H. chinensis* Qf-1; Gene name, the gene name of *H. chinensis* Qf-1; VF_ID: the gene ID information in the VF database; CARD: antibiotic resistance mechanism information in CARD data; T3SS, type III secretion system annotation information; TNSS: annotation information on the NSS secretory system.

## Data Availability

The raw sequence data reported in this paper was deposited in the Genome Sequence Archive (Genomics, Proteomics & Bioinformatics 2021), the National Genomics Data Center (Nucleic Acids Res 2022), the China National Center for Bioinformation/Beijing Institute of Genomics, and the Chinese Academy of Sciences, which are publicly accessible at https://ngdc.cncb.ac.cn/gsa, accessed on 15 May 2025. CRA025668 is the accession number of the genome data of *H. chinensis* Qf-1, while C_AA060414.1 is the accession number of the 16S rRNA gene sequence.

## Supplementary Materials

The following supporting information can be downloaded at: https://www.mdpi.com/article/10.3390/microorganisms13071604/s1, Table S1: the analysis of Average Nucleotide Identity (ANI); Table S2: the summary of TCS of *H. chinensis* Qf-1; Table S3: the summary of VFDB of H. chinensis Qf-1; Table S4: the summary of CARD of *H. chinensis* Qf-1; Table S5: the summary of T3SS of *H. chinensis* Qf-1; Figure S1: the COG annotation of *H. chinensis* Qf-1.
