# Supplementary material for: Identification and Pathogenicity Analysis of Huaxiibacter chinensis Qf-1 in Mink (Neogale vison)
Source: Microorganisms. 2025 Jul 8;13(7):1604. doi: 10.3390/microorganisms13071604 (PMC12300476; doi:10.3390/microorganisms13071604)
Supplement: Supplementary file 1 [file microorganisms-13-01604-s001.zip › Table S2 the summary of TCS of H. chinensis Qf-1.pdf]

TableS2 the summary of TCS of *H. chinensis* Qf-1

pfam_name	ORFname	pcoS	Pfam_id	E-value	score	Description
HATPase_c	chr_3374	barA	PF02518.31	2.3e-31	106.5	Histidine kinase-, DNA gyrase B-, and HSP90-like ATPase
	chr_2931	rcsC	PF02518.31	2.8e-31	106.3	Histidine kinase-, DNA gyrase B-, and HSP90-like ATPase
	chr_3908	arcB	PF02518.31	9.7e-31	104.5	Histidine kinase-, DNA gyrase B-, and HSP90-like ATPase
	chr_937	phoR	PF02518.31	5.2e-30	102.2	Histidine kinase-, DNA gyrase B-, and HSP90-like ATPase
	chr_1924	rstB	PF02518.31	4e-27	92.9	Histidine kinase-, DNA gyrase B-, and HSP90-like ATPase
	chr_3584	silS	PF02518.31	8.6e-27	91.8	Histidine kinase-, DNA gyrase B-, and HSP90-like ATPase
	chr_4330	cpxA	PF02518.31	2e-26	90.6	Histidine kinase-, DNA gyrase B-, and HSP90-like ATPase
	chr_1294	kdpD	PF02518.31	2.5e-26	90.3	Histidine kinase-, DNA gyrase B-, and HSP90-like ATPase
	chr_2790	baeS	PF02518.31	6.9e-26	88.9	Histidine kinase-, DNA gyrase B-, and HSP90-like ATPase
	chr_2140	gtrS	PF02518.31	1.2e-25	88.1	Histidine kinase-, DNA gyrase B-, and HSP90-like ATPase
	chr_3880	pmrB	PF02518.31	3.1e-25	86.8	Histidine kinase-, DNA gyrase B-, and HSP90-like ATPase
	chr_4082	envZ	PF02518.31	8.5e-25	85.4	Histidine kinase-, DNA gyrase B-, and HSP90-like ATPase

	chr_3689	qseC	PF02518.31	2.5e-24	83.9	Histidine kinase-, DNA gyrase B-, and HSP90-like ATPase
	chr_621	creC	PF02518.31	1.2e-23	81.7	Histidine kinase-, DNA gyrase B-, and HSP90-like ATPase
	chr_293	Psyr_2700	PF02518.31	1.9e-23	81.1	Histidine kinase-, DNA gyrase B-, and HSP90-like ATPase
	chr_2693	dcuS	PF02518.31	3.5e-22	77	Histidine kinase-, DNA gyrase B-, and HSP90-like ATPase
	chr_4381	ntrB	PF02518.31	1.2e-21	75.3	Histidine kinase-, DNA gyrase B-, and HSP90-like ATPase
	chr_3227	nodV	PF02518.31	1.4e-21	75	Histidine kinase-, DNA gyrase B-, and HSP90-like ATPase
	chr_3567	pcoS	PF02518.31	4.8e-21	73.3	Histidine kinase-, DNA gyrase B-, and HSP90-like ATPase
	chr_2022	phoR	PF02518.31	6.1e-21	73	Histidine kinase-, DNA gyrase B-, and HSP90-like ATPase
	chr_3158	qseE	PF02518.31	1.4e-20	71.8	Histidine kinase-, DNA gyrase B-, and HSP90-like ATPase
	chr_1456	phoR	PF02518.31	1.8e-20	71.5	Histidine kinase-, DNA gyrase B-, and HSP90-like ATPase
	chr_2538	cheA	PF02518.31	4.4e-20	70.2	Histidine kinase-, DNA gyrase B-, and HSP90-like ATPase
	chr_2944	pmrB	PF02518.31	2.2e-19	67.9	Histidine kinase-, DNA gyrase B-, and HSP90-like ATPase
	chr_4	gyrB	PF02518.31	3.8e-19	67.2	Histidine kinase-, DNA gyrase B-, and HSP90-like ATPase

	chr_1708	phoQ	PF02518.31	1.8e-18	65	Histidine kinase-, DNA gyrase B-, and HSP90-like ATPase
	chr_2392	narX	PF02518.31	2.9e-16	57.9	Histidine kinase-, DNA gyrase B-, and HSP90-like ATPase
	chr_3705	parE	PF02518.31	6.4e-15	53.6	Histidine kinase-, DNA gyrase B-, and HSP90-like ATPase
	chr_3079	narQ	PF02518.31	8.9e-15	53.1	Histidine kinase-, DNA gyrase B-, and HSP90-like ATPase
	chr_2929	rcsD	PF02518.31	4.1e-14	51	Histidine kinase-, DNA gyrase B-, and HSP90-like ATPase
	chr_35	uhpB	PF02518.31	4.8e-14	50.8	Histidine kinase-, DNA gyrase B-, and HSP90-like ATPase
	chr_902	uhpB	PF02518.31	3.2e-13	48.1	Histidine kinase-, DNA gyrase B-, and HSP90-like ATPase
	chr_3033	ypdA	PF02518.31	1.5e-12	45.9	Histidine kinase-, DNA gyrase B-, and HSP90-like ATPase
	chr_1042	htpG	PF02518.31	9e-10	37	Histidine kinase-, DNA gyrase B-, and HSP90-like ATPase
	chr_357	mutL	PF02518.31	5.5e-09	34.5	Histidine kinase-, DNA gyrase B-, and HSP90-like ATPase
	chr_2862	btsS	PF02518.31	4e-07	28.5	Histidine kinase-, DNA gyrase B-, and HSP90-like ATPase
hybrid	chr_3374	barA	PF02518.31vPF00072.29	4.3e-32	108.6	Response regulator receiver domain
	chr_2931	rcsC	PF02518.31vPF00072.29	3.3e-29	99.3	Response regulator receiver domain
	chr_3908	arcB	PF02518.31vPF00072.29	5e-23	79.4	Response regulator receiver domain
	chr_293	Psyr_2700	PF02518.31vPF00072.29	4.3e-14	50.6	Response regulator receiver domain

	chr_2022	phoR	PF02518.31vPF00072.29	2.9e-10	38.4	Response regulator receiver domain
Response_reg	chr_2525	cheY	PF00072.29	5.4e-33	111.5	Response regulator receiver domain
	chr_4382	glnG	PF00072.29	3.7e-32	108.8	Response regulator receiver domain
	chr_3374	barA	PF00072.29	4.3e-32	108.6	Response regulator receiver domain
	chr_4083	ompR	PF00072.29	7.8e-32	107.8	Response regulator receiver domain
	chr_1455	SYNPCC7002_A0851	PF00072.29	1.8e-31	106.6	Response regulator receiver domain
	chr_2139	gltR	PF00072.29	2.4e-31	106.2	Response regulator receiver domain
	chr_3156	glrR	PF00072.29	3.3e-31	105.8	Response regulator receiver domain
	chr_2573	uvrY	PF00072.29	4.7e-31	105.3	Response regulator receiver domain
	chr_4329	cpxR	PF00072.29	5.1e-31	105.2	Response regulator receiver domain
	chr_936	phoB	PF00072.29	2.6e-30	102.9	Response regulator receiver domain
	chr_2382	rssB	PF00072.29	3e-29	99.5	Response regulator receiver domain
	chr_2931	rcsC	PF00072.29	3.3e-29	99.3	Response regulator receiver domain
	chr_623	arcA	PF00072.29	5.6e-29	98.6	Response regulator receiver domain
	chr_903	uhpA	PF00072.29	1.9e-28	96.9	Response regulator receiver domain
	chr_1293	kdpE	PF00072.29	2.8e-28	96.3	Response regulator receiver domain
	chr_3583	silR	PF00072.29	4.3e-28	95.7	Response regulator receiver domain
	chr_3568	pcoR	PF00072.29	5.5e-28	95.4	Response regulator receiver domain
	chr_286	ompR	PF00072.29	1.7e-27	93.8	Response regulator receiver domain
	chr_2945	tctD	PF00072.29	4.4e-27	92.5	Response regulator receiver domain
	chr_3226	todT	PF00072.29	6e-27	92	Response regulator receiver domain
	chr_2393	narL	PF00072.29	8.4e-27	91.6	Response regulator receiver domain
	chr_2861	btsR	PF00072.29	1e-26	91.3	Response regulator receiver domain
	chr_1709	phoP	PF00072.29	1.7e-26	90.6	Response regulator receiver domain
	chr_620	creB	PF00072.29	3.5e-26	89.6	Response regulator receiver domain

	chr_34	uhpA	PF00072.29	1.1e-25	87.9	Response regulator receiver domain
	chr_3034	ypdB	PF00072.29	1.3e-25	87.7	Response regulator receiver domain
	chr_2791	baeR	PF00072.29	2.6e-25	86.8	Response regulator receiver domain
	chr_3879	pmrA	PF00072.29	3.2e-24	83.3	Response regulator receiver domain
	chr_2526	cheB	PF00072.29	1e-23	81.7	Response regulator receiver domain
	chr_3908	arcB	PF00072.29	5e-23	79.4	Response regulator receiver domain
	chr_3688	qseB	PF00072.29	8.3e-23	78.7	Response regulator receiver domain
	chr_2930	rcsB	PF00072.29	4.4e-22	76.4	Response regulator receiver domain
	chr_3228	tdiR	PF00072.29	1.2e-21	74.9	Response regulator receiver domain
	chr_1925	rstA	PF00072.29	6e-21	72.7	Response regulator receiver domain
	chr_2694	dcuR	PF00072.29	8.8e-21	72.2	Response regulator receiver domain
	chr_293	Psyr_2700	PF00072.29	4.3e-14	50.6	Response regulator receiver domain
	chr_2021	narP	PF00072.29	6.2e-14	50.2	Response regulator receiver domain
	chr_2958	cheV3	PF00072.29	2.3e-12	45.1	Response regulator receiver domain
	chr_2022	phoR	PF00072.29	2.9e-10	38.4	Response regulator receiver domain
