# Supplementary figures and images for: Identification and Pathogenicity Analysis of Huaxiibacter chinensis Qf-1 in Mink (Neogale vison)
Source: Microorganisms. 2025 Jul 8;13(7):1604. doi: 10.3390/microorganisms13071604 (PMC12300476; doi:10.3390/microorganisms13071604)

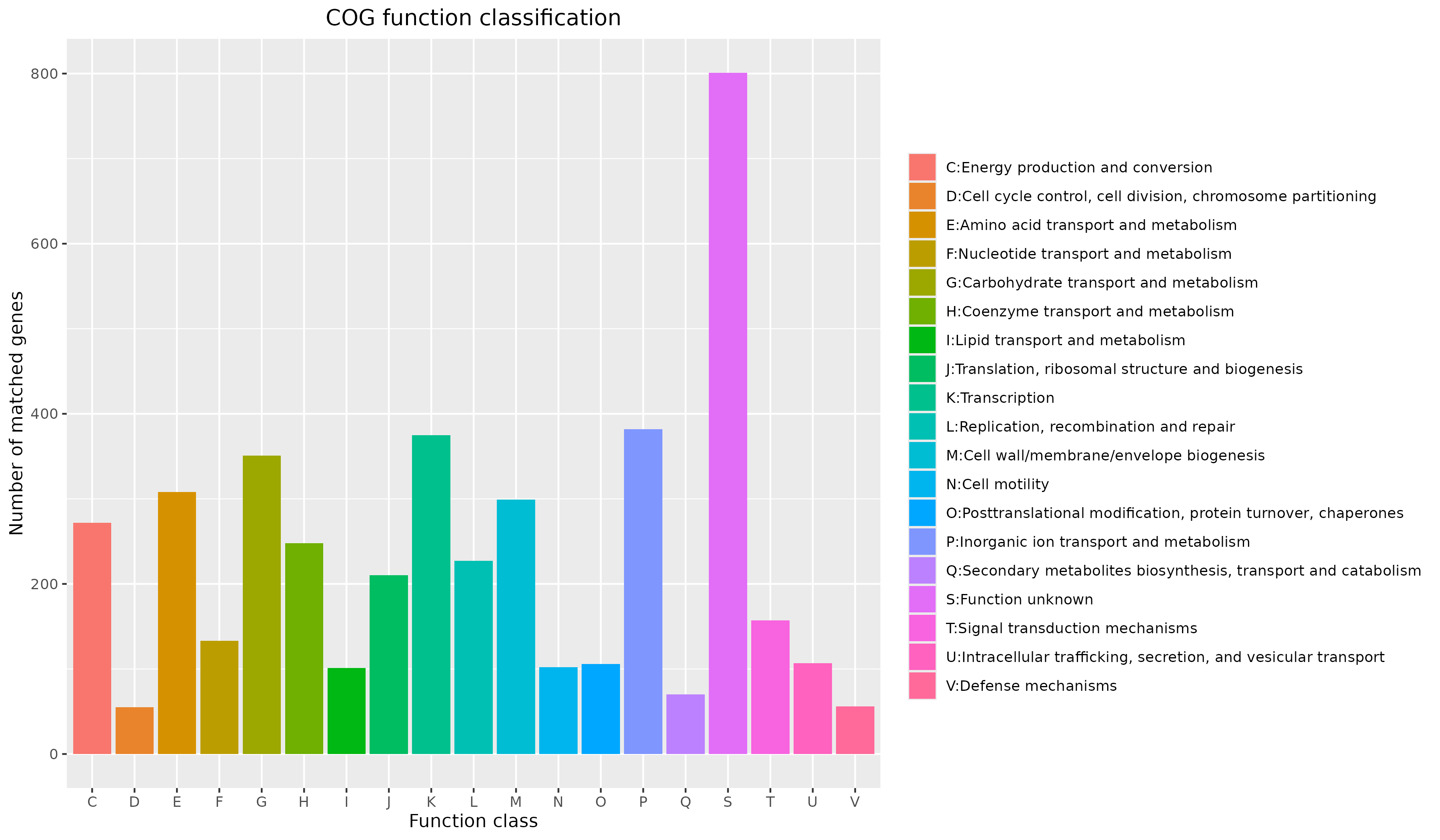

Supplement: Supplementary file 1 [file microorganisms-13-01604-s001.zip › FigureS1 the COG annotation of H. chinensis Qf-1.png]
